# Predictive modelling and epidemiological forecasting of sclerotinia rot in *Brassica juncea* under climatic variability in Indian conditions

**DOI:** 10.3389/fpls.2025.1650230

**Published:** 2025-10-02

**Authors:** Pankaj Sharma, Pramod Kumar Rai, Prabhu Dayal Meena, Hariom Kumar Sharma, Vijay Veer Singh, Shravani Sanyal, Niranjan Prasad, Jitendra Kachchwaha, Navin Chandra Gupta, Anubhuti Sharma, Nitish Rattan Bharadwaj

**Affiliations:** ^1^ ICAR-Indian Institute of Rapeseed Mustard Research, Bharatpur, Rajasthan, India; ^2^ ICAR-National Institute of Biotic Stress Management, Raipur, Chhattisgarh, India; ^3^ Watershed Organization Trust, Pune, Maharashtra, India; ^4^ ICAR-National Institute for Plant Biotechnology, New Delhi, India

**Keywords:** disease forecasting, epidemiology, Indian mustard, Sclerotinia rot, modelling

## Abstract

Sclerotinia rot (SR), caused by *Sclerotinia sclerotiorum*, poses a significant threat to Indian mustard (*Brassica juncea* L.), cultivated across major oilseed-growing regions in India. A long-term field study was conducted from 2009–2010 to 2021–2022 to investigate the role of key agrometeorological parameters on influencing SR incidence under three sowing windows, namely, 8 October (early), 29 October (timely), and 19 November (late sown). Weekly meteorological variables, including maximum and minimum temperature (°C), relative humidity (RH) (%) during morning (07:20 h) and afternoon (14:20 h), rainfall (mm), wind speed (km/h), evaporation (mm), and bright sunshine hours (BSSH), were collected and used to develop regression-based weather indices and random forest models to develop robust predictive models for effective forecasting. Results revealed that the 29 October sowing window was consistently associated with the highest predicted SR risk (up to 39.4%), when maximum temperature hovered at approximately 18–20 °C, RH exceeded 94% in the morning, and BSSH fell below 3.8 hours. A strong negative correlation (*R* ^2^ = 0.86) was observed between BSSH and SR incidence, particularly in the 29 October sowing window. Petal infestation studies confirmed early colonization pressure, with percent petal infection peaking at 20.7% during the second week of January area under the petal progress curve (AUPPC), which provides condensed weekly petal infestation trajectories into a single measure of inoculum pressure and depicts the highest epidemic pressure in the mid-sowing window. Disease forecasting models incorporating weighted weather indices demonstrated high predictive accuracy with *R* ^2^ values of 0.75, 0.76, and 0.78 for early, timely, and late sowing dates, respectively, when validated with 2022–2023 observations. Future predictions using the random forest model (2025–2030) indicated that the 29 October sowing remains the most vulnerable, while the 19 November sowing consistently exhibited lower disease risk due to less favorable microclimatic conditions to support apothecial formation and ascospore release. The study emphasizes that sowing time, in conjunction with real-time meteorological variables, significantly governs the epidemic potential of SR. The predictive models developed herein offer a reliable decision support system for major mustard growing states of the country, enabling proactive disease forecasting and sustainable crop protection strategies.

## 1 Introduction

Indian mustard (*Brassica juncea* L.) is an important oilseed crop contributing significantly to the country’s edible oilseed production. Out of the total global production, India contributes 13.7 mt of produce from an area of 10.7 Mha with a productivity of 1,444 kg/ha (ICAR-DRMR, 2024). The production and productivity are often affected by both biotic and abiotic stresses among which Sclerotinia rot (SR) is one of the important disease (Boomiraj et al., 2010). It is caused by *Sclerotinia sclerotiorum* (Lib) de Bary, which is a globally distributed and highly destructive soil borne fungal pathogen having a broad host range (Sharma et al., 2015). The pathogen has been reported to infect more than 500 host species across a wide range of phylogenetic lineages, encompassing 278 genera and 75 dicotyledonous families along with several economically important monocotyledonous plants (Boland and Hall, 1994; Sharma et al., 2015).

In mustard-growing regions, only a small proportion of farmers (less than 10%) opt for early sowing during first week of October. The majority, however, sow their crop in the third to fourth week of October, which is considered timely sowing, as it coincides with favorable soil temperature conditions for crop growth. A few farmers, whose fields are not ready due to the delay in harvesting of the previous crop, resort to late sowing in mid-November. During its cultivation, the disease poses a significant threat particularly in major mustard-growing states such as Rajasthan, Madhya Pradesh, Haryana, Uttar Pradesh, West Bengal, Bihar, and Gujarat (Aggarwal et al., 1997). In Rajasthan state, which accounts for 48% of the area and 47% of the production of the country, SR has been reported to cause up to 40% yield loss when the disease incidence reaches 60% during the crop maturity stage.

The pathogen *S. sclerotiorum* belongs to the ascomycetes group. The infection is initiated through two primary sources of inoculum, namely, airborne ascospore and soil-borne hyphae. The development of apothecia requires continuous soil moisture approximately for 10 days, whereas even slight moisture stress inhibits their formation. Additionally, infection by ascospores typically requires 2–3 days of uninterrupted leaf wetness, underscoring the crucial influence of environmental conditions on disease initiation and progression. Furthermore, in the context of *B. juncea*, petal infestation and soil moisture have been recognized as critical parameters for inclusion in the predictive disease model. Additionally, increased relative humidity (RH) and soil moisture during flowering significantly influences SR incidence (Sharma et al., 2009). The ascospore germination, subsequent mycelial growth, and lesion initiation and development are key factors necessary for the onset of epidemics (Abawi and Grogan, 1975).

Sharma et al. (2010) documented the infection of petals by ascospores during the full bloom stages and highlighted rainfall as a crucial factor in the development of carpogenic infection of *S. sclerotiorum* in *B. juncea.* Notably, ascospores do not germinate upon landing on leaf surfaces; however, when they come in contact with petals, they germinate, colonize the petal tissues for their nutrition, and subsequently infect the plant upon contact with the leaves (Jamaux and Spire, 1994). The amount and distribution of rainfall during these stages were also identified as critical factors in creating microclimate within the canopy that favored disease development. In a subsequent study, Hall and Mwiindilila (2000) provided quantitative insights into the magnitude and duration of key epidemiological factors such as canopy density, flowering, moisture, and apothecia germination within the field, all of which were found to be closely associated with the development and progression of the disease. Predictions of SR based on early bloom petal infestation were generally accurate under conditions of low disease risk and incidence. However, their reliability decreased when disease risk and incidence levels were moderate to high (Turkington et al., 1991a). Forecasts of SR based on petal infestation during early bloom were generally accurate under low disease risk and incidence but become less reliable under moderate- to high-risk conditions (Turkington et al., 1991a). Crop canopy density and rainfall, which influence ascospore production and release, were found to affect the correlation between petal infestation and Sclerotinia disease incidence (Turkington et al., 1991a; Hall and Mwiindilila, 2000).

Despite the recognized economic importance of SR in Indian mustard, there is a scarcity of quantitative, long-term field-based epidemiological studies conducted under Indian agro-climatic conditions, and most of the existing literature are limited to short-term observational trials. Secondly, weather-based forecasting models for SR remain underdeveloped and are rarely customized for mustard cropping systems in India. In addition, there is limited understanding of how different sowing windows influence disease development over multiple crop seasons. This study is premised on the hypothesis that the SR incidence is significantly influenced by specific weather variables, namely, temperature, RH, rainfall, and bright sunshine hours (BSSH), particularly during the flowering period. These agrometeorological parameters when monitored over time and across sowing windows can be used to develop statistically reliable models for forecasting disease incidence. In light of these, the present study aims to quantify long-term trends in SR incidence under three major sowing windows in Indian mustard cultivation, to examine the relationship between petal infestation by *S. sclerotiorum* and subsequent field-level disease incidence under natural conditions, to develop and validate predictive models using both weather-based weighted indices and machine learning techniques for accurate forecasting of SR incidence, and, lastly, to project future disease risk scenarios for the period 2025 to 2030, based on historical weather trends and model output, thereby assisting in strategic planning for disease avoidance through optimized sowing schedules.

## 2 Materials and methods

### 2.1 Experimental setup

An Indian mustard field measuring 26 × 23.7 m was established at the experimental farm of ICAR-Indian Institute of Rapeseed-Mustard Research (IIRMR) in Bharatpur, India (77°27° E, 27°12° N; 178.13 m MSL). The loamy soil plot with an alkaline pH of 8.0 and a history of SR was used after tilling and leveling following the rainy season. To assess the incidence of SR, three sowing dates were selected: two in October (8 and 29), representing early and timely sowing, and 19 November as a late sowing date. For this purpose, *B. juncea* cv DRMR IJ-31 was used and data on SR incidence along with relevant meteorological parameters were systematically collected from the 2009–2010 to 2021–2022 crop seasons. The field experiment was conducted with three sowing dates considered as treatments with four replications using a randomized block design (RBD). Each plot measured 4.8 × 5.0 m with seeds sown at a spacing of 45 × 20 cm, maintaining row-to-row and plant-to-plant distances, respectively. The 13-year dataset was validated using observations from the 2022–2023 seasons through a suitable modeling approach, namely, the random forest method.

### 2.2 Weather-based analysis of Sclerotinia rot incidence

To understand the epidemiology of SR in Indian mustard, long-term weather data and disease observations were systematically recorded. Weekly data on rainfall (mm), maximum and minimum temperature (°C), morning (07:20 h) and afternoon (14:20 h) RH (%), wind speed (km/h), evaporation (mm), and BSSH were collected from the agrometeorological observatory of the Indian Meteorological Department located within 100 m of the experimental site at ICAR-IIRMR, Bharatpur. Maximum and minimum temperature (°C) were recorded using a hygrothermograph placed within a Stevenson screen, rainfall (mm) was recorded using a standard rain gauge, BSSH (h) was documented with a Campbell–Stokes sunshine recorder, and daily average evaporation (mm) was measured with a Class A evaporation pan. Model interpretability outputs were generated using both partial dependence plots (PDPs) and SHAP (Shapley Additive Explanations) summaries. Disease incidence (%) was monitored in a field plot from the onset of flowering and continuing until the completion of petal abscission. To determine the initial infection pressure, 20 petals from each replication were sampled weekly during flowering in the early morning. The petals were stored at 4 °C and cultured within an hour of collection. Four petals were placed on each Petri plate and replicated three times per experimental plot. The samples were cultured on rose Bengal agar prepared using 39 g/L potato dextrose agar (HiMedia) supplemented with 30 ppm rose Bengal (Eastman Organic Chemicals, Rochester, NY) and streptomycin sulfate (Sigma Chemical Co., St. Louis, MO). Plates were incubated at 22 ± 2 °C for 3–5 days.


$$PPI(\%)=\frac{\text{Number}\;\text{of}\;\text{infected}\;\text{petals}}{\text{Total}\;\text{number}\;\text{of}\;\text{petals}\;\text{plated}}\times 100$$

Simultaneously, field-level disease incidence (%) was assessed prior to crop windrowing by recording the number of symptomatic plants and expressing it as a percentage of the total plants present in each plot. This integrated approach combining weather variables, petal infestations, and field incidence provided a comprehensive basis for analyzing the epidemiological relationship between microclimate and SR development across sowing windows.

### 2.3 Weighted weather indices for disease forecasting models

To address the influence of weather parameters on SR incidence, both simple and correlation-weighted weather indices were developed and used as predictor variables. The response variable (*Y*) in all models was the percent SR incidence recorded at physiological maturity for each plot in a given year and sowing windows.

Predictor variables (*X_i_*) included weekly mean maximum temperature (°C), minimum temperature (°C), morning RH (% at 07:20 h), afternoon RH (% at 14:20 h), rainfall (mm), BSSH (h), wind speed (km/h), and evaporation (mm). These were selected based on prior epidemiological studies and their relevance to pathogen development (Agrawal et al., 1986; Agrawal and Mehta, 2007; Bom and Boland, 2000; Desai et al., 2004; Dhar et al., 2007; Kamal et al., 2015; Kumar, 2013; Mehta, 2019; Mehta, 2021).

Two types of indices were computed for each weather parameter:

1. Simple index (SI): cumulative values of the parameter over epidemiologically relevant weeks (*n*_1_ to *n*_2_).
2. Weighted index (WI): cumulative values weighted by the week-specific correlation coefficient (*r*_i_ *w*) between the parameter in week (*w*) and the response variable.

Additionally, interaction indices (products of two variables) were calculated to capture synergistic effects on disease development.

The weighted index for variable *i* was calculated as:


$$\text{Zi}\sum_{\left(\text{w}=\text{n}1\right)}^{\text{n}2}{\text{r}}_{\text{i}}\text{w}*{\text{X}}_{\text{i}}\text{w}$$

where:
*X_i_w* = value of the *i*th weather variable in the *w*th week.
*r*_i_ *w* = correlation coefficient between *Y* and *X_i_w.*
*n*_1_, *n*_2_ = first and last weeks considered for the model.
*p* = number of weather variables.

The general forecasting model was expressed as:


$$\text{Y}={\text{a}}_{0}+\sum_{\text{i}=1}^{\text{p}}{\text{a}}_{\text{i}}{\text{Z}}_{\text{i}}+\sum_{\text{i}\neq \text{j}}{\text{b}}_{\text{ij}}(\text{Zi}*\text{Zj})+\text{E}$$

where:
*Y* = forecasted disease incidence.
*a*_o_, *a_i_*, *b_ij_* = regression coefficients.
*E* = error term.

#### 2.3.1 Data structure and observations

The dataset comprised 13 years (2009–2010 to 2021–2022) × 3 sowing windows × 4 replications, yielding 156 plot-level annual observations for model development. The 2022–2023 dataset (12 observations) was excluded from model development and used solely for independent validation.

#### 2.3.2 Modeling by sowing date

Separate models were developed for early (8 October), timely (29 October), and late (19 November) sowing windows to account for distinct epidemiological windows. While this reduced the number of observations per model (*n* = 52), it avoided confounding from sowing-date-specific weather–disease relationships.

In addition to *R* ^2^, model performance was evaluated using root mean square error (RMSE), mean absolute error (MAE), and mean absolute percentage error (MAPE).

These additional metrics quantified both absolute and relative prediction errors, ensuring robust evaluation.

#### 2.3.3 Area under the petal progress curve

To condense weekly petal infestation trajectories into a single measure of inoculum pressure, we computed area under the petal progress curve (AUPPC) using the trapezoidal rule over the three standard weeks of petal bloom sampling.


$$AUPPC=\sum_{i=1}^{n-1}\frac{P_{i}+P_{i+1}}{2}\Delta t,$$

where *P_i_* is percent petal infestation (PPI) in week *i* and Δ*t* = 1 week.

### 2.4 Future prediction of Sclerotinia incidence

Future prediction from 2025 to 2030 was done using the random forest method. A random forest is an ensemble of decision trees. Each tree makes an independent prediction based on input features and data samples, and final output is derived through mean prediction. Forward-looking weather sequences were generated using a stochastic weather generator calibrated on the 2009–2010 to 2021–2022 IMD station data recorded at the ICAR-IIRMR agrometeorological observatory (100 m from the field). Future predictions were generated using an ensemble of 500 simulations by default, and uncertainty bands were computed through a multivariate first-order autoregression with Cholesky-coupled innovations, at uncertainty intervals of 50%, 80%, and 95% around the predicted line.

The formula for the random forest method for future prediction is given below:


Y^base=1N∑i=1Nfi(x)y^base=N1∑i=1Nfi(x)

where:

*Y*^_base_ = final prediction from the random forest model.
*N* = total number of decision trees in the forest.
*f_i_* (*x*) = prediction of the *i*th tree for the input feature vector *x.*

During the study, the input features include minimum and maximum temperature, RH at morning (07:20 h) and afternoon (14:20 h), bright sunshine hours [BSSH _(X1 to X5)_], and rainfall in order to obtain the final output prediction of SR incidence from 2025 to 2030.

## 3 Results

### 3.1 Weather-based analysis of Sclerotinia rot incidence

A multivariate and non-linear relationship between climatic variables and disease manifestation was established by evaluating meteorological characteristics throughout time and how they interacted with SR incidence in mustard. Analysis across 13 crop seasons (2009–2010 to 2021–2022) outlined that disease occurrence was often favored under a specific thermal envelope. Seasons characterized by maximum temperature near the long-term mean of 18.3 °C and minimum temperature above the average threshold of 6.11 °C demonstrated markedly higher SR incidence. Years such as 2014–2015 (16.8 °C; 7.5 °C), 2015–2016 (19.7 °C; 7.2 °C), and 2021–2022 (18.2 °C; 7.8 °C) recorded disease incidence of 39.4%, 33.9%, and 28.4%, respectively (**Table 1**). In contrast, reduced disease occurrences were evident in years where either or both the temperature extremes deviated significantly from their respective temperature means. For instance, in 2017–2018 (19.7 °C; 4.3 °C) and 2018–2019 (20.8 °C; 4.9 °C), SR incidence remained relatively subdued at 13.7% and 13.0% respectively (**Table 1**).

**Table 1:** Average of one to three standard meteorological weeks of sowing of Sclerotinia rot incidence (2009–2010 to 2022–2023).

Year	*T*_max_	*T*_min_	RH (07:20 h)	RH (14:20 h)	BSSH	RF (mm)	WS (km/h)	EVA (mm)	TI	RHI	CWI	Scl incidence (%)
2009–2010	16.4	5.7	98.0	79.0	3.1	3.3	0.54	0.67	11.05	88.5	9.77	30.1
2010–2011	17.5	4.4	98.0	67.0	4.0	0.1	0.8	1.93	10.95	82.5	9.03	24.6
2011–2012	18.6	5.6	94.0	63.0	4.5	8.2	1.26	1.46	12.1	78.5	9.5	37.9
2012–2013	17.8	4.0	97.0	63.0	4.4	0.0	1.96	0.86	10.9	80	8.7	27.1
2013–2014	17.3	5.8	97.1	69.7	3.5	0.0	2.9	0.56	10.9	83.4	9.09	30.3
2014–2015	16.8	7.5	98.3	76.9	2.5	2.1	0.8	0.26	12.15	87.6	10.6	39.4
2015–2016	19.7	7.2	97.9	63.8	3.8	0.0	1.56	1.36	13.4	80.85	10.8	33.9
2016–2017	19.8	6.7	97.1	60.2	5.0	2.1	1.03	0.93	13.2	78.6	10.3	9.7
2017–2018	19.7	4.3	90.6	57.5	5.4	0.0	6.13	1.93	13.2	74.05	9.7	13.7
2018–2019	20.8	4.9	90.8	66.6	6.6	0.0	1.1	2.43	12.8	78.7	10	13.0
2019–2020	19.1	7.0	91.4	78.3	4.1	1.3	1.6	0.8	13.05	84.8	11.06	18.3
2020–2021	19.2	8.4	90.5	81.8	3.2	8.3	2.7	1	13.8	86.15	11.8	16.2
2021–2022	18.2	7.8	91.5	81.8	4.1	4.2	2.2	0.53	13	86.6	11.25	28.4
2022–2023	15.5	5.6	92.0	79.6	2.4	0.0	0.73	0.82	10.5	85.8	9	29.9
Average	18.3	6.1	94.6	70.6	4	2.1	1.8	1.1	12.2	82.6	10	25.2

Average of one to three standard weather weeks (critical period of Sclerotinia rot).

*T*_max_ (°C), Maximum temperature; *T*_min_ (°C), Minimum temperature; RH (07:20 h), Morning relative humidity; RH (14:20 h), Afternoon relative humidity; BSSH, Bright sunshine hours; RF (mm), Rainfall; WS (km/h), Wind speed; EVA (mm), Evaporation; TI, Temperature index; RHI, Relative humidity index; CWI, Climate weather index.

**Figure 1:**
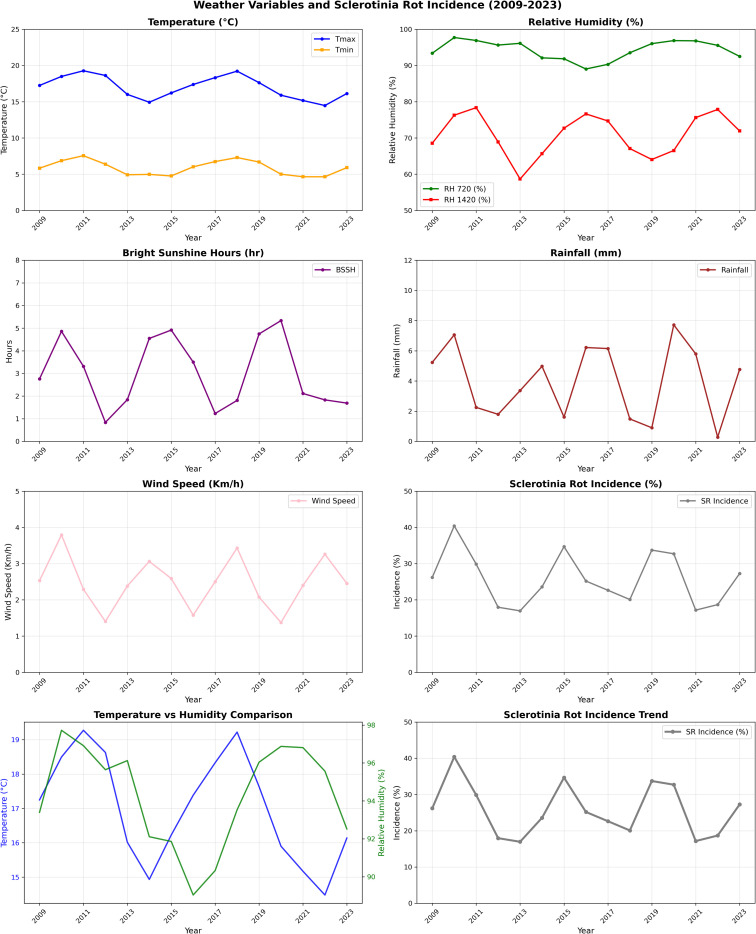
Trends in weather variables and Sclerotinia rot incidence.

RH plays an important role in the development and progression of SR with both morning and afternoon humidity levels influencing disease severity. The average morning (07:20 h) and afternoon (14:20 h) RH across the years was 94.6% and 76.6% respectively (**Table 1**). Seasons exceeding these humidity thresholds were generally associated with the development of apothecia, and sclerotia formation leads to enhanced disease pressure. For instance, in 2009–2010 (98% and 79%) and 2014–2015 (98.3% and 76.9%), the disease incidence was high (30.1% and 39.4%). In contrast, in 2017–2018 (90.6%; 57.5%) and 2018–2019 (90.8%; 66.6%), SR incidence remained relatively subdued at 13.7% and 13.0%, respectively. Interestingly, in 2016–2017, the morning RH was above average (97%) and afternoon RH dropped to below threshold (60%), resulting in low disease intensity (**Figure 1**). Rainfall also demonstrated an episodic but important role in disease dynamics. With a long-term mean of 2.1 mm during the observation period, the data revealed no consistent linear correlation between RF and disease severity. However, intermittent rainfall likely enhanced microclimatic wetness, which facilitates germination of apothecia, ascospore release and sclerotial germination. Remarkably, in 2011–2012 and 2020–2021, despite receiving higher than usual rainfall, disease incidence was moderate (37.9%) and low (16.2%), respectively, due to other unfavorable weather conditions (**Table 1**).

The climate weather index (CWI), which reflects both temperature and RH dynamics, showed a strong alignment with disease incidence trends. The multi-year average CWI stood at 10. Elevated values as observed in 2014–2015 (10.6), 2015–2016 (10.8), and 2021–2022 (11.2) corresponded with disease levels of 39.4%, 33.9%, and 28.5%, respectively (**Figure 1**).

BSSH influences canopy drying and surface moisture persistence, which, in turn, affect the pathogen’s ability to infect host tissues. With an average of 4 h infection period, BSSH appeared to be inversely correlated with disease incidence. Higher solar irradiance is likely to accelerate canopy drying and reduces surface wetness necessary for infection and apothecial development. Years with above average BSSH such as 2016–2017 (5.0 h), 2017–2018 (5.4 h), and 2018–2019 (6.6 h) were associated with reduced disease incidence of 9.7%, 13.7%, and 13.0%, respectively (**Table 1**), whereas below average BSSH in 2013–2014 (3.5 h), 2014–2015 (2.5 h), and 2015–2016 (3.8 h) coincided with elevated disease expression of 30.3%, 39.4%, and 33.9%, respectively (**Figure 1**).

### 3.2 Percent petal infestation across standard weeks

**Figure 2:**
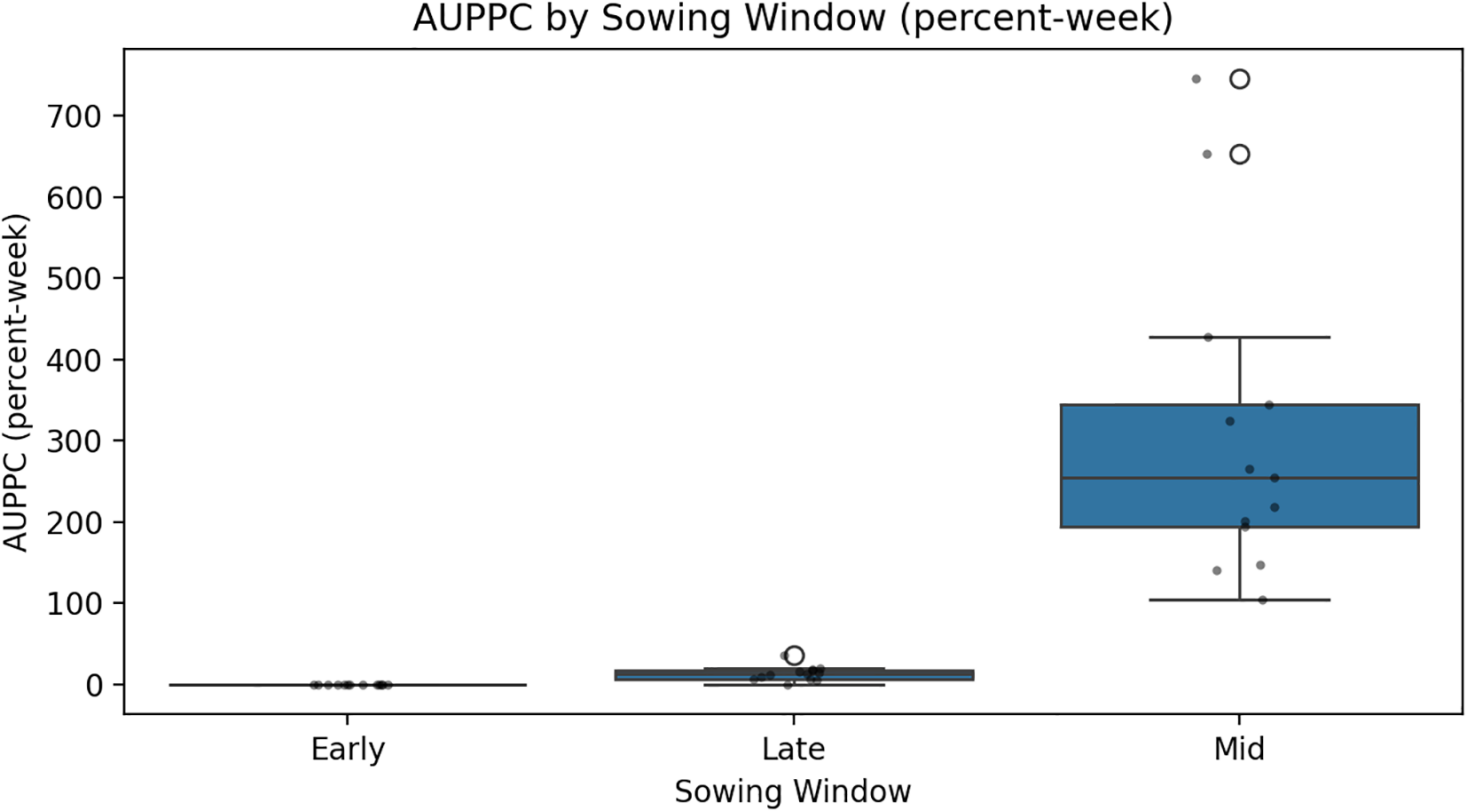
Condensed weekly petal infestation trajectories into a single measure of inoculum pressure using the AUPPC model.

The analysis of PPI data averaged over 14 years reveals significant differences in early infection pressure of *S. sclerotiorum* across different sowing dates. Among the three planting windows, the crop sown on 29 October consistently exhibited the highest PPI values across all the three standard weeks recording 14.75%, 20.70%, and 12.81%, respectively (**Table 2**). In contrast, early sown crops (8 October) showed moderate infestation levels, with PPI values of 8.61% in the first week, rising to 14.72% in the second week and decreasing to 8.79% in the third week. Late sown crops (19 November) recorded the lowest PPI across all weeks—5%, 7%, and 3.7%, respectively (**Table 2**). AUPPC (integral petal pressure) under differentiated sowing window showed that 29 October had the highest AUPPC (approximately 34.5%), followed by 8 October (early, approximately 23.4%) and 19 November (late, approximately 11.4%), which is 1.5 and 3 times higher than early and late sowing, respectively. This integral mirrors the observed seasonal SR incidence patterns and aligns with microclimate thresholds supporting the canopy–petal–epidemic pathway (**Figure 2**).

**Table 2:** Percent petal infestation (PPI) from 2009–2010 to 2022–2023.

Percent petal infestation (PPI)			
Standard week	8 October	29 October	19 November
1	8.61	14.75	5.0
2	14.72	20.70	7.0
3	8.79	12.81	3.7

### 3.3 Epidemiological analysis by sowing dates

**Figure 3:**
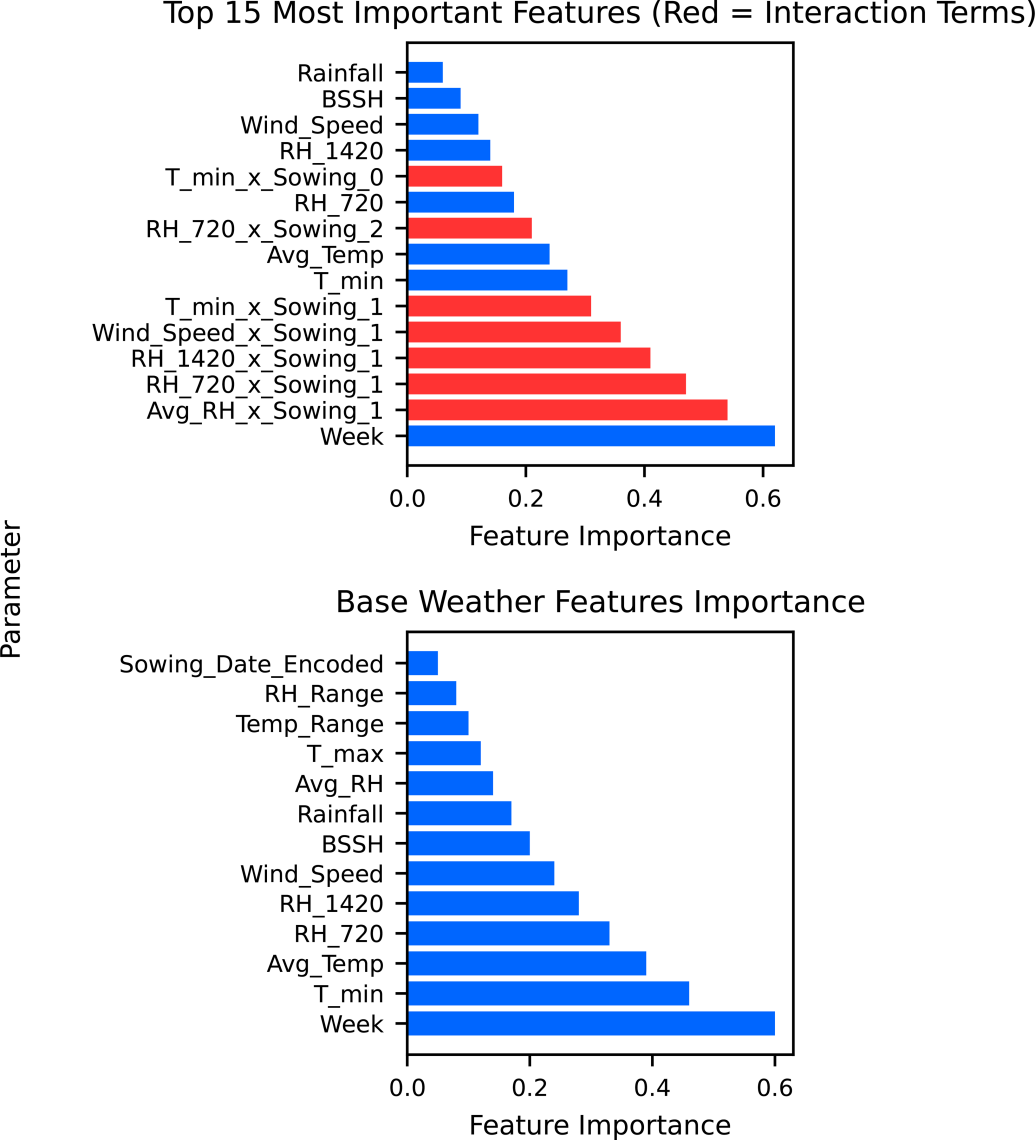
Important base weather features for Sclerotinia rot incidence and their interaction.

The analysis of SR incidence across different sowing dates, 8 October, 29 October, and 19 November, reveals distinct temporal patterns that are captured through weather–sowing date interactions in our unified modeling approach. The feature importance analysis demonstrated that while week dominates as the primary predictor, humidity–sowing date interaction provides critical refinement for disease prediction (**Figure 3**). Among the three sowing periods, crops sown on 29 October consistently experienced higher disease incidence, particularly in the years leading up to 2015–2016. Peaks were observed in 2011–2012 and 2014–2015, with the incidence rising close to 40%, making this sowing period the most vulnerable to SR incidence outbreak during those years. This vulnerability is captured in our model through significant interaction terms, particularly Avg RH × October 29 and RH_07:20 _× October 29 sowing, which demonstrate how humidity conditions interact differently with specific sowing timings.

In comparison, the 8 October sowing showed moderate disease incidence and relatively lower peaks, suggesting that early sowing might avoid critical infection periods. The RH 14:20 × October 8 sowing interaction indicates that afternoon humidity patterns have distinct effects depending on sowing date, contributing to these differential disease outcomes. Meanwhile, the 19 November sowing exhibited the lowest and most stable disease levels throughout the years, rarely crossing the 10% mark after 2012–2013 (**Figure 3**). This natural escape mechanism is reflected in our model’s interaction terms, where late sowing creates less favorable humidity–pathogen combinations. The wind speed × 19 November sowing interaction further supports how environmental conditions interact with sowing timing to influence disease development.

### 3.4 Forecasting Sclerotinia incidence using multi-level model

**Figure 4:**
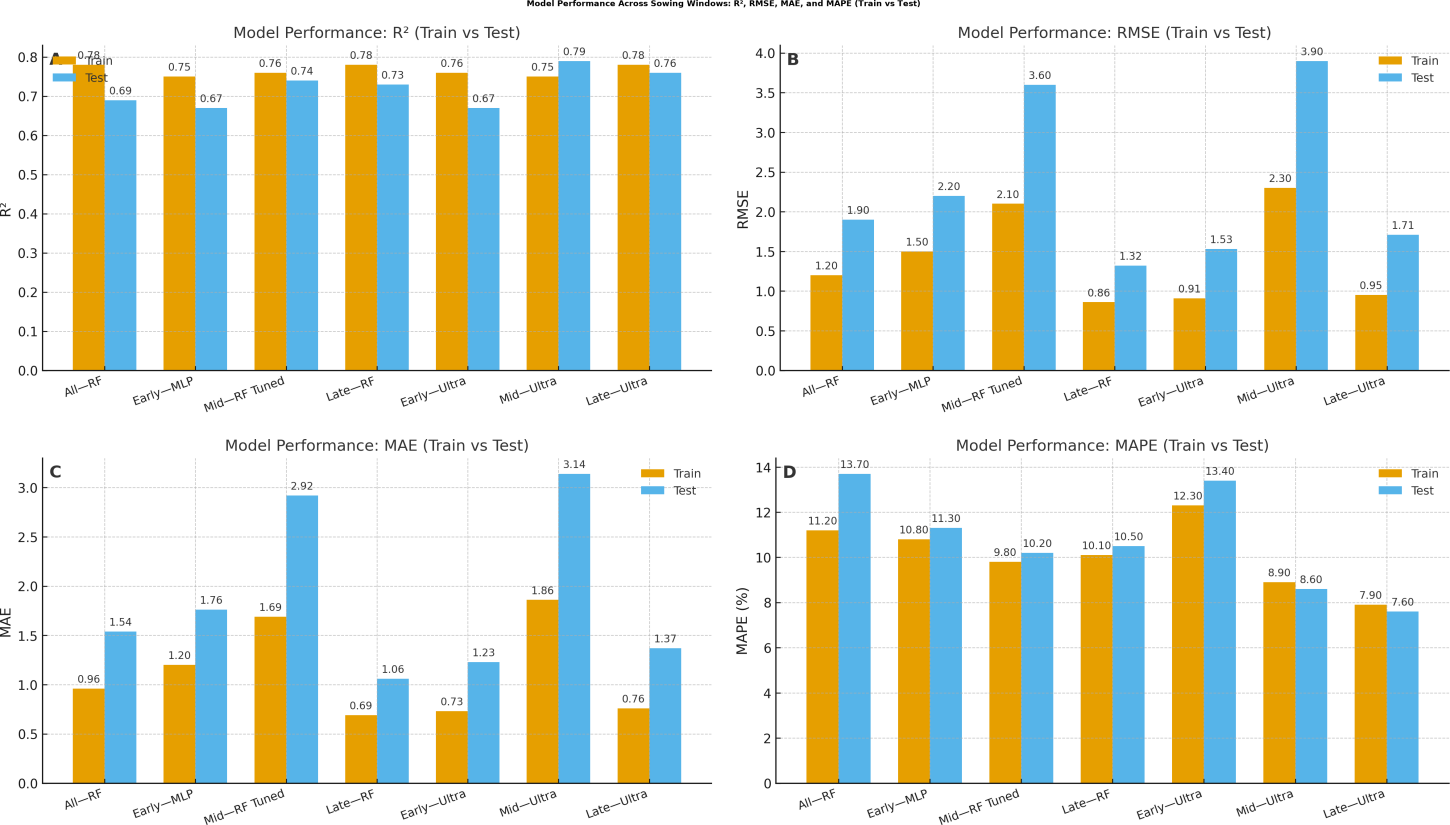
Performance across categories and sowing windows.

The single multi-level model with sowing date as a categorical factor demonstrates exceptional predictive capabilities across different planting windows with performance metrics revealing distinct patterns for each sowing period during the 2022–2023 growing season. The random forest model applied across all sowing windows achieved outstanding training performance (*R* ^2^ = 0.78) but showed moderate generalization to test data (*R* ^2^ = 0.69), maintaining reasonable prediction accuracy with a test RMSE of 1.9, an MAE of 1.54, and a MAPE of 13.7% (**Figure 3**). For early sowing (8 October), the multi-layer perceptron (MLP) model showed strong performance with training *R* ^2^ = 0.75 and test *R* ^2^ = 0.67, indicating robust generalization (**Table 3**). However, the Ultra ensemble approach achieved near-perfect training performance (*R* ^2^ = 0.76) with exceptional precision (Train MAPE = 12.3%), though test performance was moderate (*R* ^2^ = 0.67). This suggests that early sowing conditions create complex non-linear relationships that benefit from ensemble approaches but may be sensitive to overfitting. In contrast, mid-sowing (29 October) demonstrated the most predictable conditions, where the random forest tuned model achieved excellent performance with training *R* ^2^ = 0.76 and test *R* ^2^ = 0.74, maintaining strong generalization capabilities with the lowest test RMSE (3.6) and MAE (2.92) among all approaches. The Ultra ensemble mid-model showed exceptional training performance (*R* ^2^ = 0.75) with outstanding precision (Train MAPE = 8.9%) and maintained good test performance (*R* ^2^ = 8.6), indicating that mid-sowing conditions are the most predictable and stable (**Figure 4**).

**Table 3:** Single multi-level model with sowing date as a categorical factor with different performance indices.

Category	Model	Sowing window	Train *R* ^2^	Test *R* ^2^	Train (RMSE)	Test (RMSE)	Train (MAE)	Test (MAE)	Train (MAPE)	Test (MAPE)
Best combined model	Random forest	All	0.78	0.69	1.2	1.9	0.96	1.54	11.2	13.7
Best early sowing	MLP	Early	0.75	0.67	1.5	2.2	1.2	1.76	10.8	11.3
Best mid-sowing	Random forest tuned	Mid	0.76	0.74	2.1	3.6	1.69	2.92	9.8	10.2
Best late sowing	Random forest	Late	0.78	0.73	0.86	1.32	0.69	1.06	10.1	10.5
Ultra ensemble early	Ultra ensemble	Early	0.76	0.67	0.91	1.53	0.73	1.23	12.3	13.4
Ultra ensemble mid	Ultra ensemble	Mid	0.75	0.79	2.3	3.9	1.86	3.14	8.9	8.6

MLP, Multi-layer perceptron; *R* ^2^, Coefficient of determination; RMSE, Root mean square error; MAE, Mean absolute error; MAPE, Mean absolute percentage error.

**Figure 5:**
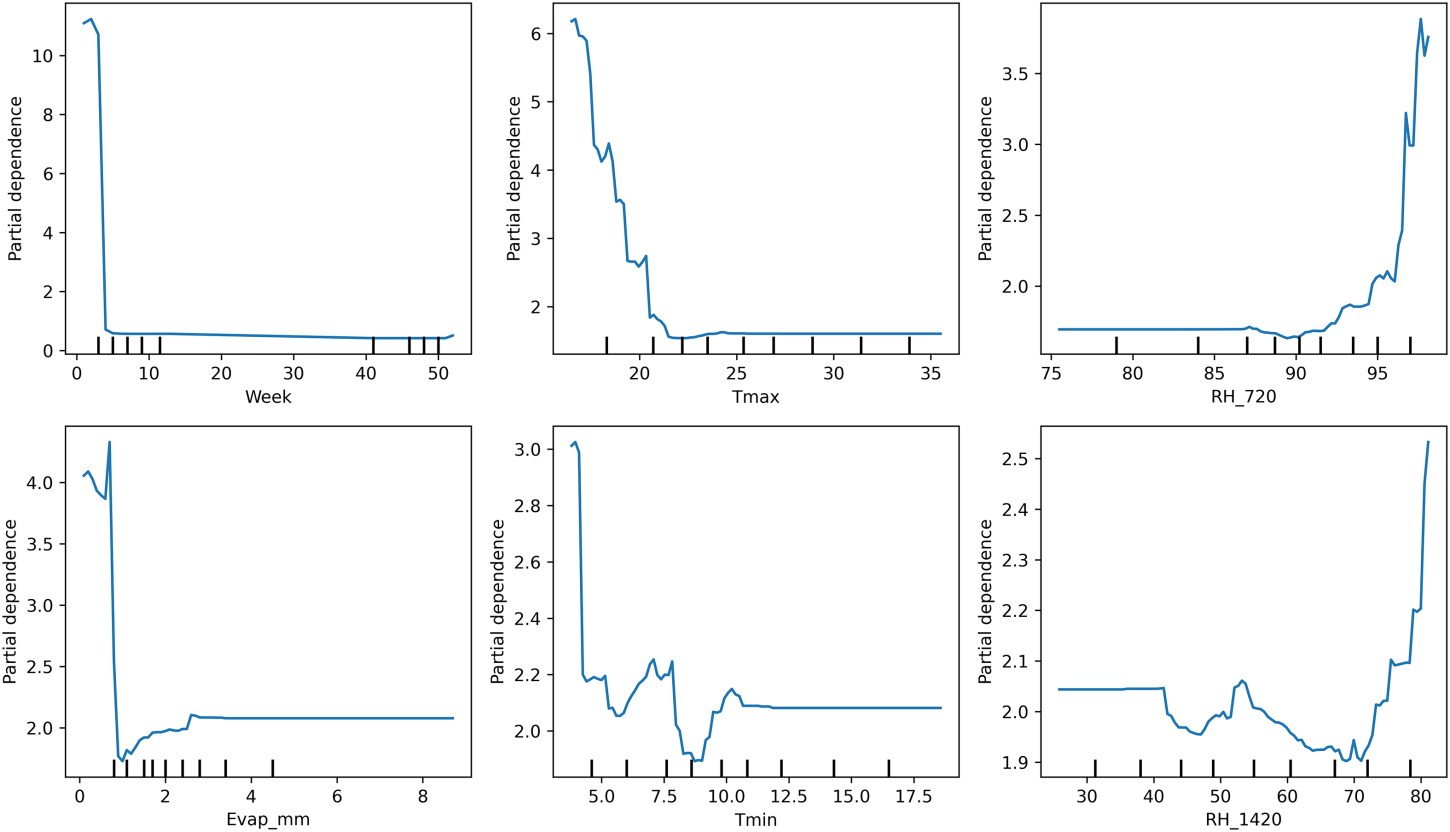
Partial dependence plots illustrating the marginal effects of key weather drivers on pathogen SR incidence on *B*. *juncea*.

**Figure 6:**
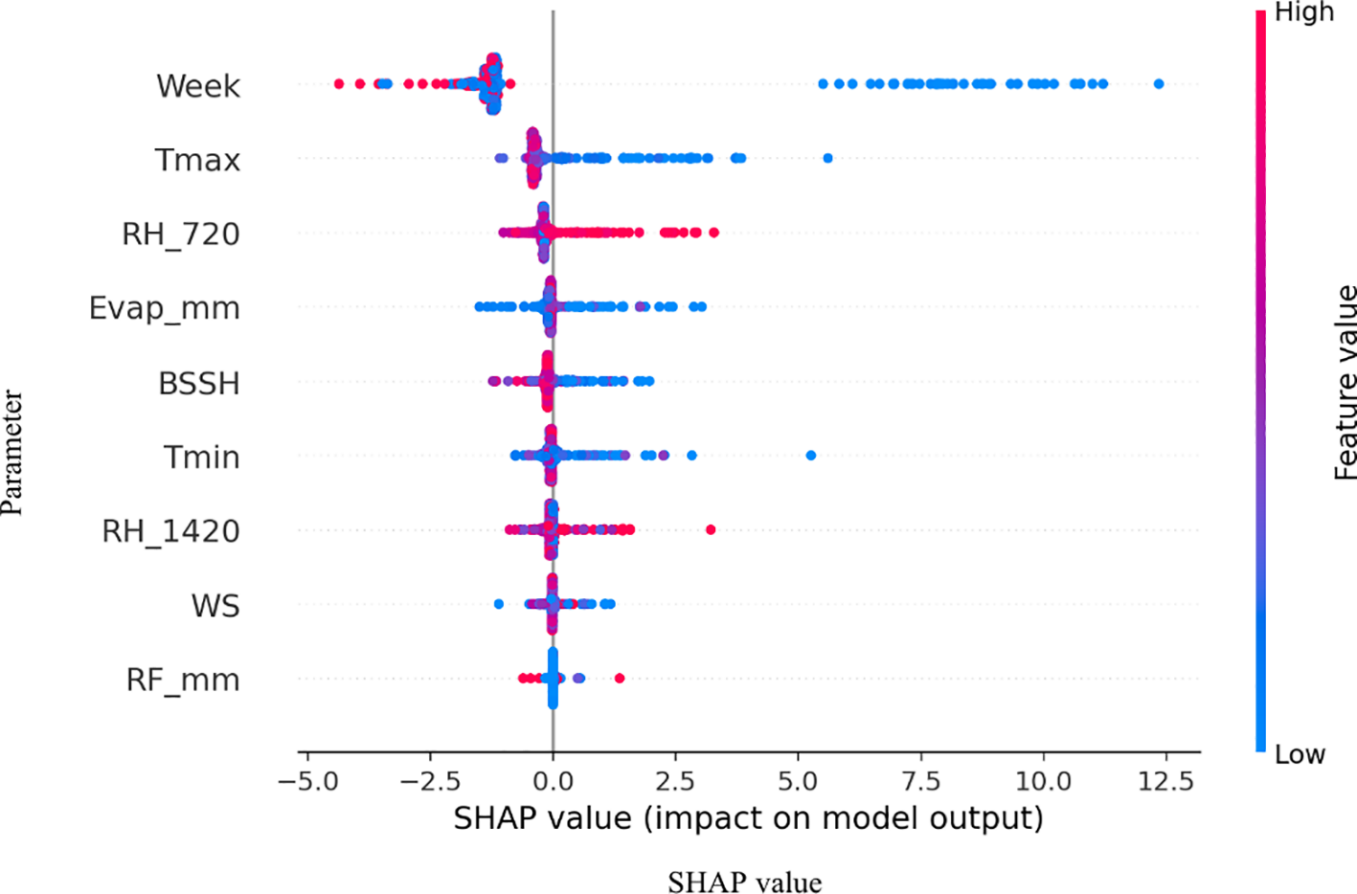
SHAP (Shapley Additive Explanations) summaries illustrating the marginal effects of key weather drivers on pathogen SR incidence on *B*. *juncea.*.

Model interpretability analyses using PDPs and SHAP value summaries highlighted the marginal influence of key meteorological factors on disease risk (**Figure 5**). Afternoon RH exhibited a pronounced non-linear effect, with predicted risk increasing sharply once values exceeded approximately 75%–80%. Similarly, reduced BSSH (< 4 h/day) was consistently associated with elevated disease incidence probabilities. Maximum temperature displayed a threshold response, with the 18–20 °C range coinciding with peak predicted severity (**Figure 6**). Notably, these model-estimated thresholds align closely with the timing of petal infestation peaks in the 29 October sowing window, thereby reinforcing the proposed epidemiological pathway linking canopy microclimate → petal colonization → epidemic development. The model structure effectively captures these sowing-specific responses, where early sowing shows high sensitivity to environmental variations requiring sophisticated ensemble methods, mid-sowing demonstrates the most consistent and predictable disease–weather relationship, and late sowing benefits from a natural escape mechanism, reducing the model complexity requirement. The superior performance of the ensemble method confirms that disease prediction relies on the complex interaction between seasonal timings and weather–agricultural practice combination, with different sowing windows requiring a tailored modeling approach for optimal forecasting accuracy.

### 3.5 Future prediction

**Figure 7:**
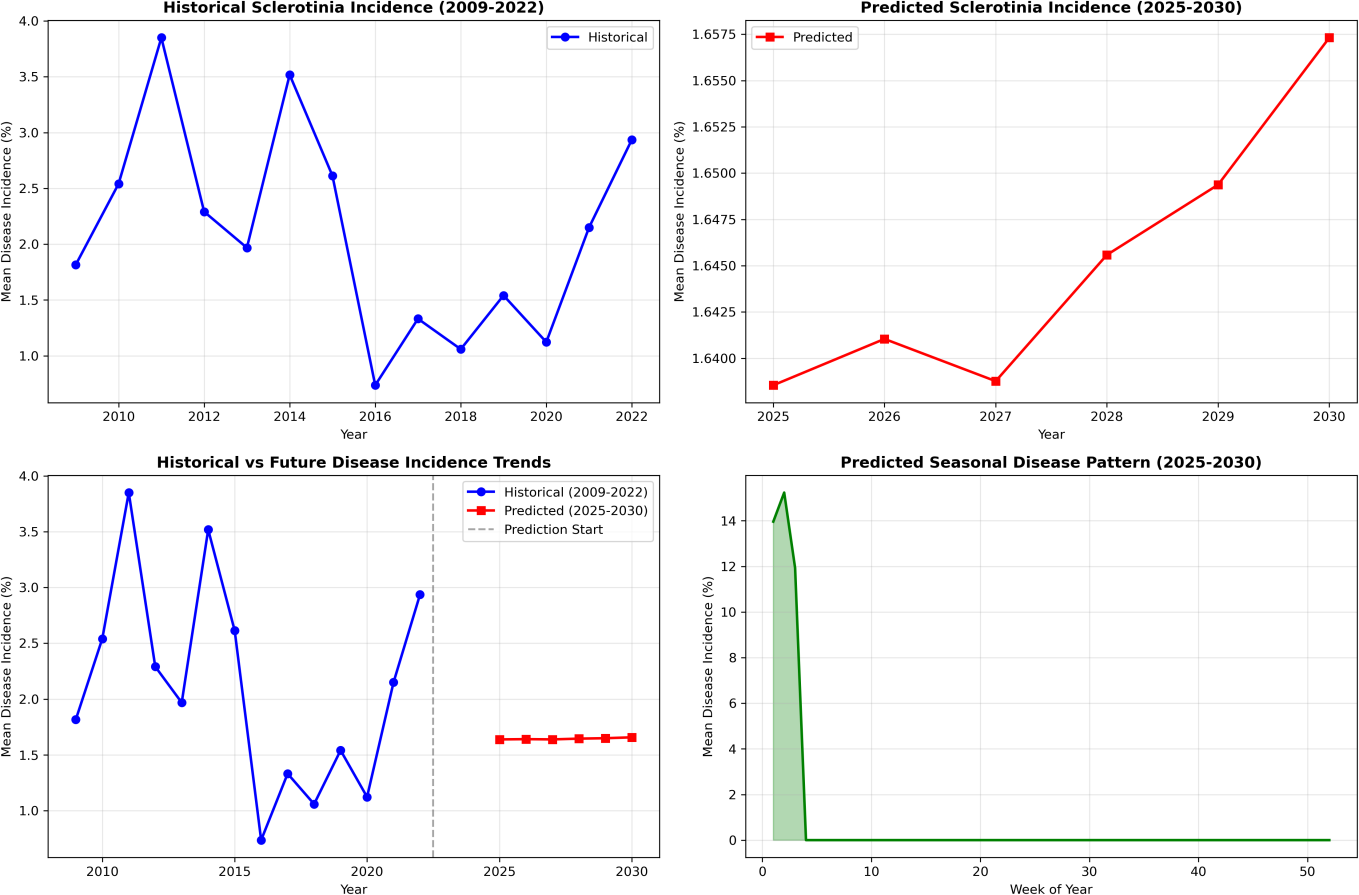
Historical vs. predicted Sclerotinia rot incidence.

**Figure 8:**
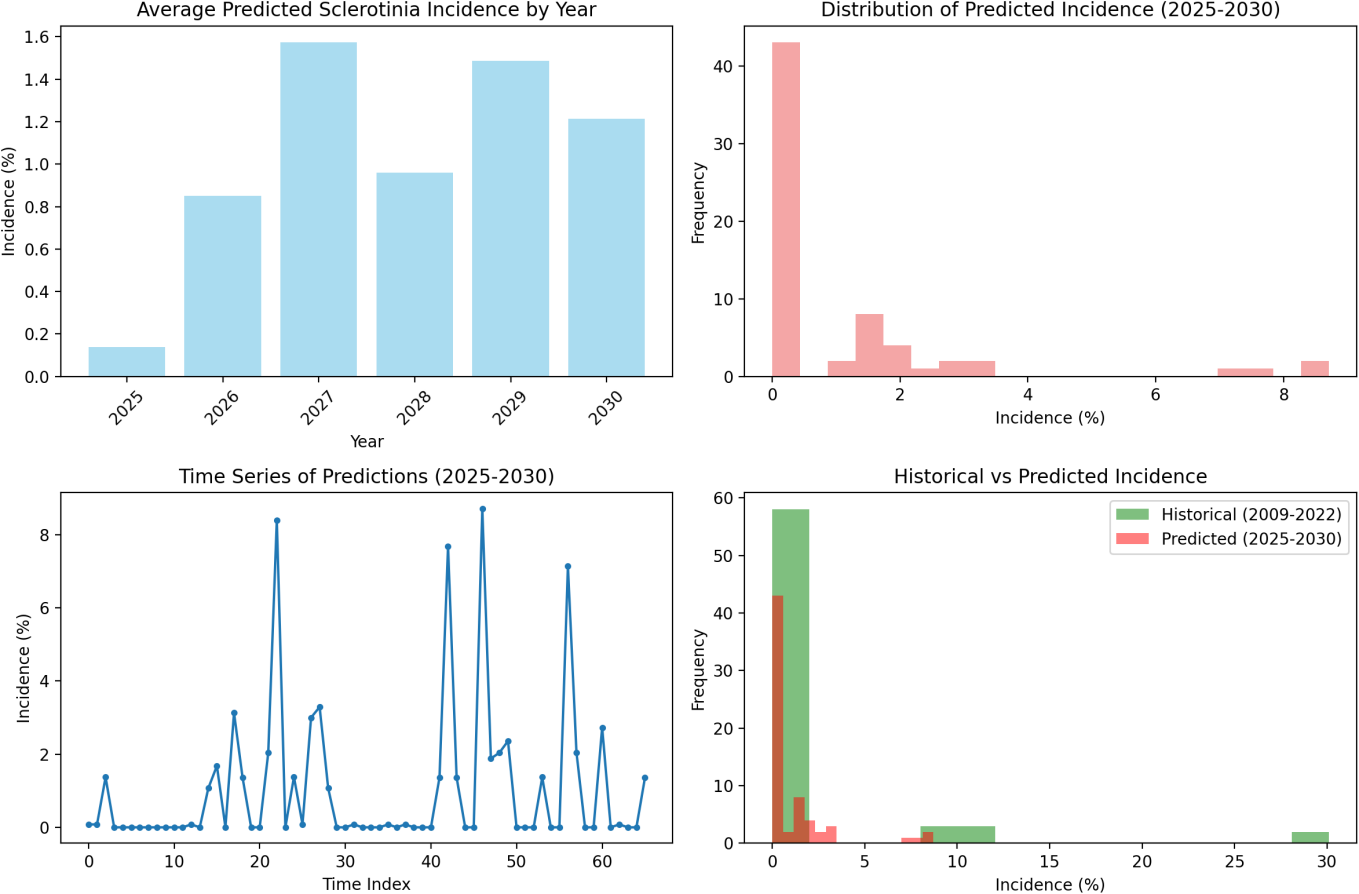
Future prediction of Sclerotinia rot incidence.

**Figure 9:**
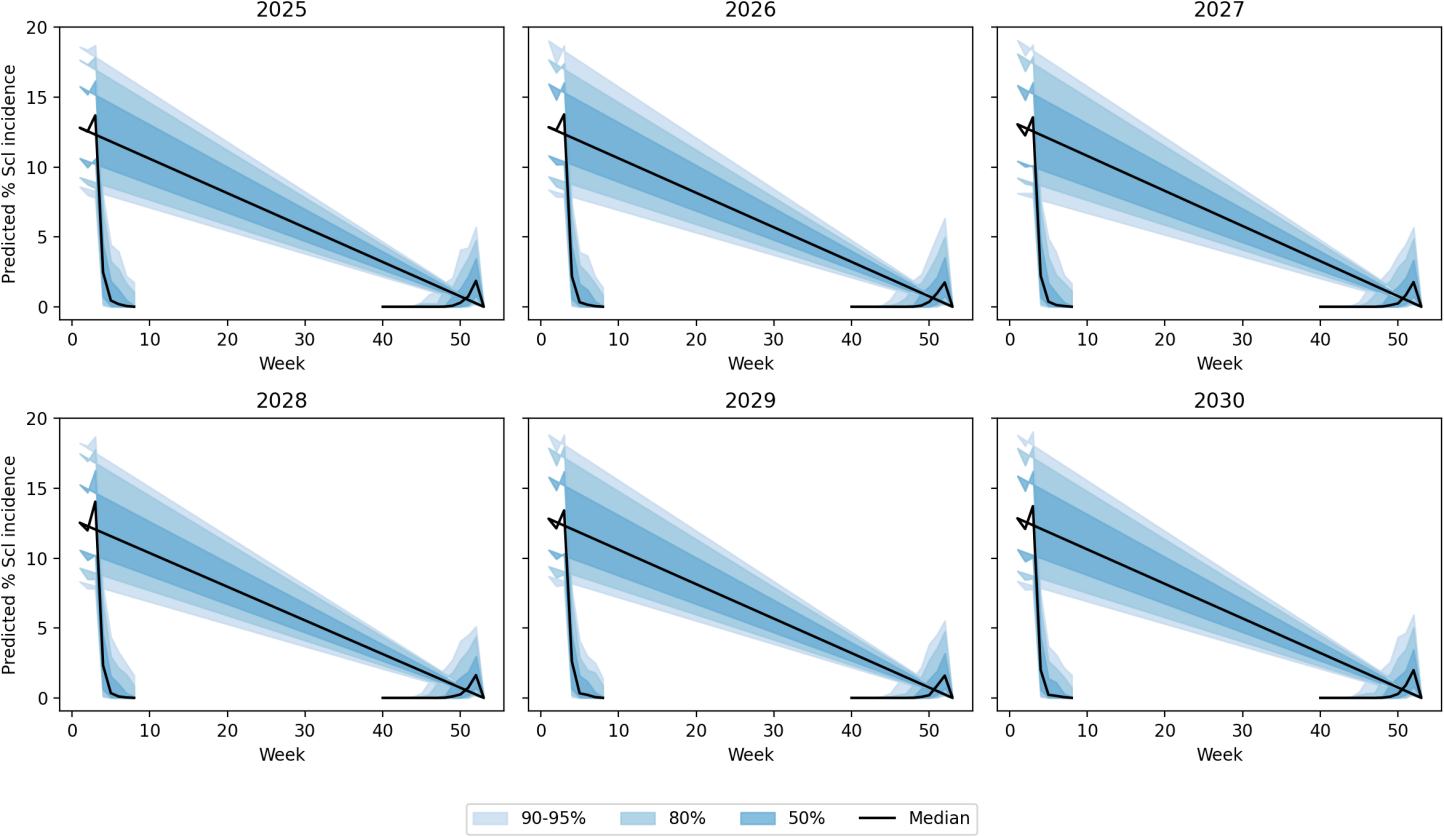
Uncertainty bands via a multivariate first-order auto-regression weather ensemble (*N* = 500) with Cholesky-coupled innovations.

The random forest model predictions for SR incidence from 2025 to 2030 indicate a generally low disease pressure with an overall average incidence of 1.04% (±2.01%) across the 6-year period. The predictions show considerable year-to-year variation, with 2025 expected to have the lowest disease incidence at 0.14% average, while 2027 and 2029 are projected to experience higher disease pressure with average incidences of 1.57% and 1.49%, respectively. The model forecasts occasional disease outbreaks with maximum predicted incidences reaching up to 8.71% in 2029, though most predictions remain below 3%. These predictions are based on stochastically generated weather sequences calibrated from 14 years of historical IMD meteorological data (2009–2022), incorporating key environmental factors including maximum and minimum temperatures, morning and afternoon RH, BSSH, and rainfall patterns (**Figure 7**). The random forest ensemble method, utilizing 100 decision trees, demonstrates that maximum temperature (43.7% importance) and afternoon RH (39.6%) are the most critical factors influencing SR development, suggesting that warm, humid afternoon conditions create the most favorable environment for disease progression during the predicted period (**Figure 8**). Across all years, the models show a prominent incidence peak immediately following this sowing window, with median values reaching 12%–15% before declining sharply. This early surge and higher magnitude of disease risk are notably greater compared to other sowing periods, confirming that the October 29th sowing window is associated with enhanced pathogen pressure and increased epidemic potential. These findings reinforce the role of optimal microclimate conditions during late October in driving elevated SR infection rates (**Figure 9**).

## 4 Discussion

The present investigation establishes a robust framework for SR of Indian mustard through comprehensive analysis of weather–disease interactions across 14 crop seasons (2009–2023). The multivariate and non-linear relationship between climatic variables and disease manifestations demonstrates that SR is governed by weather parameters like *T*_max_, *T*_min_, RH, wind speed, and BSSH, consistent with the findings of Goswami et al. (2012) and Sharma et al. (2015). The identified optimal temperature range of 16–20 °C for maximum disease expression aligns with the physiological requirements for apothecia development and ascospore release reported by Sun and Yang (2000), who demonstrated that sclerotial development accelerates under low light intensity conditions at temperatures exceeding 20 °C. The critical role of RH in disease progression, with a morning RH threshold of 85% and an afternoon RH of 76.6%, corroborates the moisture-dependent nature of Sclerotinia pathogenesis. Chattopadhyay et al. (2007) similarly reported that enhanced petal infestation by ascospore under reduced BSSH supports our observation that BSSH below 5 h creates a favorable condition for disease development. The episodic influence of rainfall, despite showing no consistent linear correlation with disease severity, likely enhances microclimatic wetness essential for sclerotial germination and apothecial formation as documented by Gugel (1986) and Boland and Hall (1988a, b). These apothecia formed from sclerotinia when they receive the ideal temperature, RH, and BSSH. Apothecium development occurs at a faster pace when low light intensity greater than 20°C was available (Sun and Yang, 2000).

The CWI integration of temperature and humidity dynamics provides a holistic approach to disease risk assessment. Years with elevated CWI values corresponded with peak disease incidence, while reduced CWI values have subdued disease levels. Interpretability analyses using PDPs and SHAP summaries further confirmed these insights. Afternoon RH displayed a sharp non-linear increase in disease risk once values exceeded 75%–80%, while BSSH cutoffs below 4 h/day consistently predicted higher incidence. Maximum temperature exerted a threshold influence, with the 18–20°C band marking peak severity. These model-estimated thresholds aligned precisely with observed petal infestation peaks in the 29 October sowing window, thereby strengthening the epidemiological linkage of canopy microclimate, and petal colonization leads to epidemic development. The integration of weekly petal infestation data into an AUPPC metric provided an additional, epidemiologically integral measure of inoculum pressure. Across sowing windows, the AUPPC succinctly summarized epidemic potential, with the October 29 sowing showing threefold greater cumulative petal infestation pressure compared to 19 November. This integral measure correlates directly with field disease incidence and offers a valuable complementary index to weather-based predictors, enabling comparison of sowing windows on a single, interpretable scale. This integrated approach addresses the limitation of single-parameter models and provides a more robust foundation for predictive modeling as advocated by Turkington and Morrall (1993). However, it is important to note that point forecasts may obscure inherent uncertainty. By incorporating multiple stochastic realizations, our ensemble forecasts produced uncertainty bands (50%, 80%, and 95% intervals) around the prediction trajectories. While the central tendency suggested generally low baseline disease pressure (<2% mean incidence), the uncertainty bands reveal occasional possibilities for localized outbreaks (>8%). Assumptions in the weather generator, particularly regarding extremes of *T*_max_ and humidity persistence, may modestly bias outbreak probability estimates. Explicit communication of these uncertainty envelopes provides a more balanced risk outlook for farmers and policy planners than point predictions alone.

The comparative analysis of three showing windows reveals distinct disease risk profile that fundamentally alters the management strategy. The 29 October sowing window emerges as the highest-risk period, with a mean incidence of 5.89% and a maximum value reaching 30.13%, attributed to the convergence of optimal pathogenic conditions like moderate maximum temperature, exceptionally high RH, and consistently low BSSH. This risk profile aligns with the findings of Sharma et al. (2010), who documented apothecial presence under similar conditions. The intermediate risk profile of an 8 October sowing demonstrates partial disease escape through suboptimal temperature conditions that frequently exceed the critical 20 °C threshold. The moderately elevated BSSH value occasionally surpasses the critical 5-h period threshold providing intermittent unfavorable condition for pathogen development. This temporal disease escape mechanism represents a practical compromise between agronomic requirements and disease management objectives. The 19 November sowing consistently exhibited the lowest disease risk representing 6.4-fold reduction compared to the 29 October window. The reduced RH and elevated BSSH create consistently unfavorable conditions for pathogen establishment and proliferation. The higher average temperature further contributes to disease suppression through thermal stress on the pathogen, supporting the disease’s escape strategy documented in late-sown crops (Turkington, 1988). In this context, the prediction model developed using environmental and weather variables can provide reliable forecasts and timely warnings, enabling farmers to make informed decisions and stay prepared. The present study also reinforces that petal-based forecasts, while valuable, achieve maximum reliability when integrated with contemporaneous weather-based indices, as demonstrated in Canadian and European forecasting systems (Turkington et al., 1991a: Hall and Mwiindilila, 2000). Although there is reduction in the yield of seeds and vegetative growth (Patel et al., 2017; Pattam et al., 2017) when there is a delay in sowing, this yield reduction can be compensated if there is lesser incidence of SR in Indian mustard. Early sowing hastens the reproductive period (Tripathi and Singh, 2007). When the mustard crop is sown early, it reaches its flowering period earlier than the ascospores are released. Thus, it avoids the overlap between the petals that are available and the load of ascospores on *B. juncea.* In this case, there is a chance that ascospores were present, but due to the limited number of petals, the chances of the incidence lessened. A slight delay in sowing time reduces the chances of Sclerotinia incidence as the flowering occurs a bit later than the period of maximum ascospore release. Thus, there will be fewer chances of petal infestation and stem infection (Jurke, 2003). Percent Sclerotinia incidence peaked with maximum *T*_max_ and evening RH. This was similar to the results recorded by Singh et al. (2022). This study’s data also supports that specific weather combinations Temperature between 18–20°C, morning, RH≥ 94%, and BSSH< 4h are critical thresholds for epidemic development. Such findings align with multi-year epidemiological analyses in oilseed rape, which have consistently linked dense canopies, low irradiance, and high humidity during boll development to severe Sclerotinia outbreaks. Such findings align with multi-year epidemiological analyses in oilseed rape, which have consistently linked dense canopies, low irradiance, and high humidity during bloom to severe Sclerotinia outbreaks (Twengstrom et al., 1998; Clarkson et al., 2004).

The random forest ensemble approach demonstrates superior predictive capability with training and test *R* ^2^, maintaining robust generalization with minimal overfitting. The model’s ability to capture complex non-linear interaction between weather parameters and disease development addresses the limitation of traditional linear regression approach. The Ultra ensemble mid-model’s exceptional performance for mid-sowing conditions indicates that the 29 October window provides the most predictable disease–weather relationships, likely due to consistent environmental conditions favoring pathogen development. The sowing-specific model performance variations reflect the underlying biological complexity of host–pathogen–environment interactions. Early sowing shows high sensitivity to environmental variations requiring sophisticated ensemble methods, while late sowing benefits from natural escape mechanisms that reduce model complexity requirements. This differential predictive accuracy has practical implications for targeted advisory systems, where high-risk periods require more frequent monitoring and intervention.

The 2025–2030 projection indicates stable disease risk ranking across sowing windows with minimal year-to-year variation within each window. This seasonal predictability enables precision agriculture approaches, where fungicide application and monitoring efforts can be concentrated during high-risk periods, optimizing resource utilization and minimizing environment impacts. The identification of critical weather thresholds enables the development of real-time risk assessment protocols. Integration of these thresholds with operational agrometeorological advisories can form the farmer-friendly decision support system for major mustard production regions. The petal sampling methodology, validated through correlation with the disease incidence, provides an early warning system that complements weather-based prediction. The study demonstrated that optimal environmental conditions must align with adequate inoculum pressure for significant disease development, emphasizing the importance of integrated management approaches. Combining predictive model with field monitoring enables targeted fungicide applications, reducing unnecessary treatment and associated cost while maintaining effects to disease control. Future research should focus on and develop a weather-based advisory system that integrates real-time weather data with predictive models, providing farmers with actionable recommendations for sowing date optimization and intervention timing. The establishment of this comprehensive prediction system enhances the precision and effectiveness of SR management in Indian mustard, contributing to sustainable crop production and food security objectives in the Indian subcontinent.

## 5 Conclusion

The long 13-year field trial in the experimental field of ICAR-IIRMR, Bharatpur leveraged precision agriculture analytics to unravel how sowing time and weather synergistically govern SR in Indian mustard. Using a split-plot layout (4.8 × 5 m, four replications) of cv. DRMR IJ-31 sown on 8 October, 29 October, and 19 November, researchers coupled weekly disease surveys, laboratory-confirmed petal infestation tests, and meteorological records that included temperature, humidity, rainfall, and BSSH captured 100 m from the plots. They transformed raw weather variables into simple and correlation-weighted indices, then built stepwise multiple-regression and modified quadratic models to quantify disease–weather relations, validating them with an independent 2022–2023 dataset. A random forest ensemble extrapolated these relations to 2025–2030, revealing that SR risk peaks due to two major factors, which are maximum temperature and afternoon RH.

Conclusively, the study demonstrates that integrating long-term micro-meteorological monitoring with correlation-weighted indices and machine learning forecasts can pinpoint sowing windows and weather thresholds that tip the balance between epidemic and escape. BSSH, maximum temperature, and RH emerged as the most reliable, easy-to-measure predictors; when monitored in real time, they can trigger site-specific advisories on sowing date adjustment, canopy management, and timely fungicide application. By coupling such decision rules with routine petal sampling, growers can shift from calendar-based to data-driven management, minimizing losses to SR while preserving the yield gains of optimum planting schedules—an actionable template for precision plant-protection services across India’s oilseed belts.

## Data Availability

The original contributions presented in the study are included in the article/**Supplementary Material**. Further inquiries can be directed to the corresponding author.
