# Supplementary figures and images for: Predictive modelling and epidemiological forecasting of sclerotinia rot in Brassica juncea under climatic variability in Indian conditions
Source: Front Plant Sci. 2025 Oct 2;16:1650230. doi: 10.3389/fpls.2025.1650230 (PMC12528185; doi:10.3389/fpls.2025.1650230)

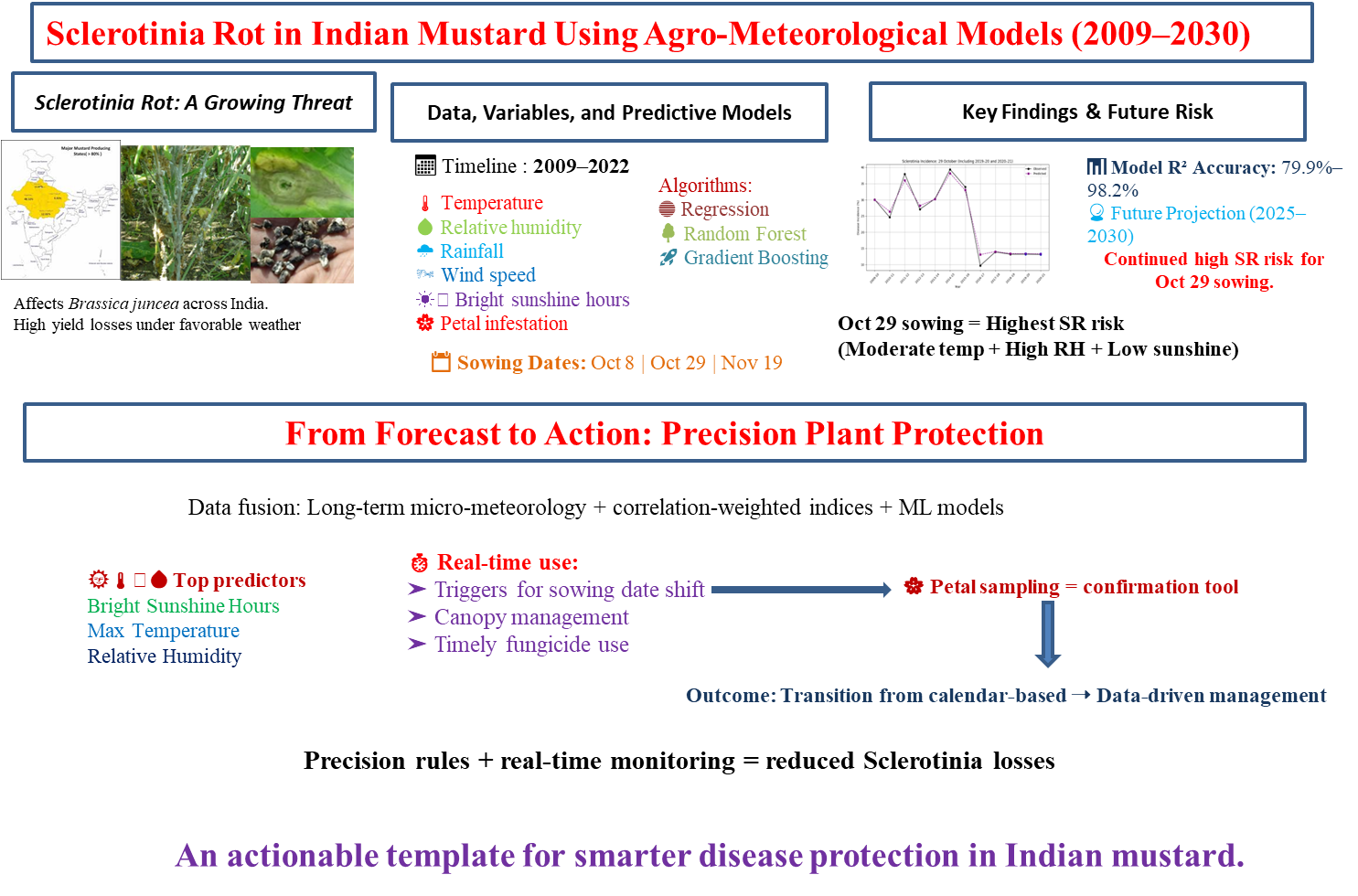

Supplement: Supplementary file 1 [file Image1.png]
